# Quantum GHZ Multiplexer: Hierarchical Teleportation for 1→2*^n^* Quantum Networks

**DOI:** 10.3390/e28050529

**Published:** 2026-05-07

**Authors:** Luis Adrián Lizama-Pérez

**Affiliations:** Departamento de Sistemas de Información y Comunicaciones, Universidad Autónoma Metropolitana, Av. de las Garzas 10, El Panteón, Lerma de Villada 52005, Mexico; l.lizama@correo.ler.uam.mx

**Keywords:** quantum routing, GHZ states, quantum teleportation, quantum networks, hierarchical quantum communication

## Abstract

We introduce a quantum multiplexer (GHZ MUX) architecture that enables deterministic routing of an unknown qubit from a single sender to one of $2^{n}$ receivers using only local tripartite Greenberger–Horne–Zeilinger (GHZ) states arranged in a binary tree. At each level of the hierarchy, a Bell-basis measurement and classical feed-forward propagate the encoded quantum information along a selected branch while maintaining the appropriate Pauli correction frame. Unlike quantum routing architectures that rely on globally entangled multipartite states, the proposed design composes small GHZ clusters into a modular teleportation hierarchy that requires only local entanglement generation and coherence. This structure achieves full input–output connectivity while preserving deterministic routing control and experimental feasibility for near-term small-scale quantum networks. Beyond routing functionality, we show that the same GHZ-tree structure naturally supports hidden-destination communication. We formalize this extension as the *Hidden-Secret GHZ-Tree Routing (HS-GTR)* protocol, in which the final receiver remains unknown to external observers and the transmitted quantum state may optionally be protected by a quantum one-time pad. This construction demonstrates that hierarchical GHZ routing can serve not only as a quantum switching architecture but also as a building block for privacy-preserving communication and multi-receiver key establishment in distributed quantum networks.

## 1. Introduction

Quantum teleportation is a cornerstone process in quantum information science, enabling the faithful transfer of an arbitrary quantum state |X〉=α|0〉+β|1〉 (where ${\left|\alpha \right|}^{2}+{\left|\beta \right|}^{2}=1$) between distant nodes by means of pre-shared entanglement and classical communication [1]. The protocol, first proposed by Bennett et al., has been experimentally realized across multiple physical platforms, including photonic systems [2], trapped ions [3], and superconducting qubits [4,5]. More recently, teleportation has been demonstrated in hybrid quantum systems and metropolitan-scale fiber networks [6,7], establishing the operational foundation of quantum communication and paving the way toward the quantum internet.

Among the pioneering theoretical contributions, Cirac et al. [8] introduced the concept of *entanglement swapping*, a mechanism that extends the range of teleportation across linear networks through intermediate nodes sharing Bell pairs. Subsequent research has refined this principle through efficient entanglement-swapping protocols optimized for multi-user quantum networks [9]. Building upon these developments, Pant et al. [10] proposed the *quantum router*, a protocol capable of distributing quantum information in coherent superposition to multiple receivers using multipartite GHZ states. This idea significantly expanded the design space of quantum network topologies, enabling dynamic routing and parallel entanglement distribution, further supported by recent advances in multipartite routing algorithms and GHZ-state generation [11,12,13].

Recent advances have further underscored the importance of multipartite entanglement as a resource in near-term quantum networking. For example, the optimized distribution of general graph-state entanglement in network settings was recently addressed (see, e.g., Fan et al. 2024 [14]) in which generation and routing of complex entangled topologies across heterogeneous infrastructures were studied. Modular network architectures that rely on locally coherent entangled clusters—rather than globally entangled states—have also been proposed as scalable blueprints for quantum-communication infrastructure (e.g., Sen et al. 2023 [15]). These developments collectively support the view that GHZ- and graph-state-based strategies may present practical near-term alternatives to fully coherent global-router models.

Within this framework, the proposed *GHZ MUX* generalizes the concept of quantum teleportation by enabling the deterministic transfer of a quantum state |X〉 from a single emitter to one of $2^{n}$ receivers through a hierarchical structure of local tripartite GHZ states, $\frac{1}{\sqrt{2}}\left(|000\rangle +|111\rangle \right)$. This arrangement forms a binary tree of entanglement, where each level performs a teleportation step mediated by Bell-basis measurements and classical communication. The device achieves full connectivity—any input can reach any output—while maintaining localized entanglement clusters, making it functionally analogous to a classical multiplexer switch but implemented with quantum resources.

### Contributions of This Work

The main contribution of this work is the introduction of a hierarchical quantum routing architecture based on local tripartite GHZ resources. Rather than relying on globally entangled multipartite states or on linear chains of Bell-pair entanglement swapping, the proposed *GHZ multiplexer (GHZ MUX)* organizes small GHZ clusters into a binary entanglement tree that enables deterministic transfer of a quantum state from a single emitter to one of $2^{n}$ receivers.

Operationally, each internal node of the tree performs a Bell-basis measurement that propagates the encoded quantum information toward a selected subbranch, while classical feed-forward communication maintains the appropriate Pauli correction frame along the route. This mechanism provides full input–output connectivity while preserving strictly local entanglement generation and coherence requirements.

Conceptually, the GHZ MUX should therefore be understood not as a new teleportation protocol, but as a *quantum routing architecture* that composes elementary GHZ-mediated transfer blocks into a scalable binary switching structure. This perspective differentiates the GHZ MUX from quantum routers based on globally entangled multipartite states [10] and from entanglement-swapping networks constructed solely from Bell pairs [8]. The resulting design is modular, locally coherent, and therefore particularly suitable for near-term implementations of small- to medium-scale quantum communication networks.

Beyond deterministic routing, the GHZ MUX architecture also supports communication primitives in which the effective destination of the quantum state remains hidden from external observers. This capability is formalized in the *Hidden-Secret GHZ-Tree Routing (HS-GTR)* protocol presented in the Appendix A, which exploits the same hierarchical GHZ-tree structure to enable destination-hiding communication and multi-receiver key-establishment scenarios in distributed quantum networks.

Finally, this work complements our recent Bell-basis computational framework, where structured bipartite entanglement was explored as an operational primitive for intrinsic verification in quantum processing [16]. While that approach focuses on computation and error detectability through Bell correlations, the present manuscript extends these ideas to communication settings by employing hierarchical GHZ entanglement as a routing resource.

The remainder of this paper is organized as follows. Section 2 describes the operational principles of the GHZ MUX, including both the base configuration and its recursive generalization. Section 3 presents a comparative analysis with the *Quantum Switch* of Cirac et al. and the *Quantum Router* of Pant et al., focusing on connectivity, resource efficiency, and coherence requirements. Section 4 discusses the practical limitations of the architecture and outlines possible directions for future developments. Finally, Section 5 summarizes the main conclusions of this work. For completeness, Appendix A introduces the Hidden-Secret GHZ-Tree Routing (HS-GTR) protocol, which illustrates how the same hierarchical GHZ structure can support destination-hiding communication and multi-receiver key-establishment primitives.

## 2. Basic Operation Mode of the GHZ MUX

The GHZ MUX operates by teleporting a quantum state |X〉 from an emitter (Alice) to one of $2^{n}$ possible receivers ($R_{1},\ldots ,R_{2^{n}}$) through a hierarchical arrangement of $2^{n}-1$ local tripartite GHZ states. Each GHZ state serves as a quantum communication channel linking either an emitter, an intermediate node, or a receiver. The network can thus be visualized as a binary entanglement tree, where each node performs a Bell-basis measurement that transfers the quantum information downward until it reaches the designated output node. This process combines quantum teleportation with classical routing control, achieving a deterministic mapping between inputs and outputs. The fundamental mechanism is best understood by first analyzing the simplest configuration (n=1), followed by its recursive generalization.

### 2.1. Base Case: MUX from 1 to 2

**Figure 1:**
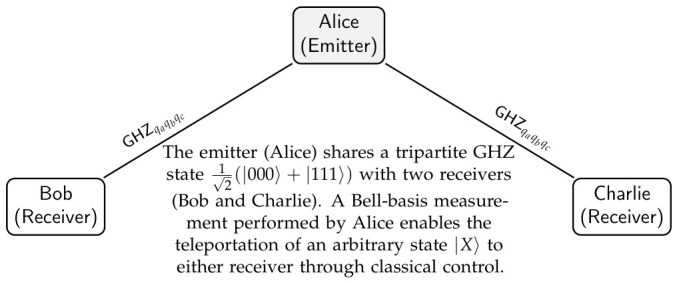
Schematic representation of the GHZ MUX (base case, n=1). The emitter (Alice) teleports an arbitrary quantum state to one of two receivers (Bob or Charlie) using a single tripartite GHZ state.

Consider the elementary configuration of the GHZ MUX in which Alice (*A*) intends to teleport an arbitrary qubit |X〉 to one of two receivers: Bob (*B*) or Charlie (*C*). This simplest 1→2 GHZ MUX configuration is illustrated schematically in Figure 1. Alice holds a qubit $q_{x}$ in the unknown state ${|X\rangle }_{q_{x}}=\alpha |0\rangle +\beta |1\rangle$ and a second qubit $q_{a}$, which forms part of a shared tripartite GHZ state:

$${|\mathrm{GHZ}\rangle }_{q_{a}q_{b}q_{c}}=\frac{1}{\sqrt{2}}\left(|000\rangle +|111\rangle \right),$$

where $q_{b}$ belongs to Bob and $q_{c}$ to Charlie. The aim is to teleport |X〉 to either $q_{b}$ or $q_{c}$, depending on the classical control signal sent after Alice’s measurement.

The initial combined system is:

$$|\psi_{0}\rangle ={|X\rangle }_{q_{x}}⊗{|\mathrm{GHZ}\rangle }_{q_{a}q_{b}q_{c}}={\left(\alpha |0\rangle +\beta |1\rangle \right)}_{q_{x}}⊗\frac{1}{\sqrt{2}}{\left(|000\rangle +|111\rangle \right)}_{q_{a}q_{b}q_{c}}.$$

Alice performs a Bell-basis measurement on her two qubits $q_{x}$ and $q_{a}$, defined by the states:

(1) $$\begin{array}{cccc} |\Phi^{+}\rangle & =\frac{1}{\sqrt{2}}\left(|00\rangle +|11\rangle \right), & |\Phi^{-}\rangle & =\frac{1}{\sqrt{2}}\left(|00\rangle -|11\rangle \right), \end{array}$$

(2) $$\begin{array}{cccc} |\Psi^{+}\rangle & =\frac{1}{\sqrt{2}}\left(|01\rangle +|10\rangle \right), & |\Psi^{-}\rangle & =\frac{1}{\sqrt{2}}\left(|01\rangle -|10\rangle \right). \end{array}$$

After expanding $|\psi_{0}\rangle$ and rewriting it in the Bell basis for $q_{x},q_{a}$, we obtain:

(3) $$\begin{array}{c} |\psi_{0}\rangle =\frac{1}{2}[{|\Phi^{+}\rangle }_{q_{x}q_{a}}(\alpha {|0\rangle }_{q_{b}}{|0\rangle }_{q_{c}}+\beta {|1\rangle }_{q_{b}}{|1\rangle }_{q_{c}})\\ \;+{|\Phi^{-}\rangle }_{q_{x}q_{a}}\left(\alpha {|0\rangle }_{q_{b}}{|0\rangle }_{q_{c}}-\beta {|1\rangle }_{q_{b}}{|1\rangle }_{q_{c}}\right)\\ \;+{|\Psi^{+}\rangle }_{q_{x}q_{a}}\left(\alpha {|1\rangle }_{q_{b}}{|0\rangle }_{q_{c}}+\beta {|0\rangle }_{q_{b}}{|1\rangle }_{q_{c}}\right)\\ \;+{|\Psi^{-}\rangle }_{q_{x}q_{a}}\left(\alpha {|1\rangle }_{q_{b}}{|0\rangle }_{q_{c}}-\beta {|0\rangle }_{q_{b}}{|1\rangle }_{q_{c}}\right)]. \end{array}$$

Each Bell outcome occurs with equal probability 1/4. Suppose first that Alice’s Bell-basis measurement yields ${|\Phi^{+}\rangle }_{q_{x}q_{a}}$. Then the conditional state of Bob and Charlie becomes

$$|\psi_{BC}^{\left(\Phi^{+}\right)}\rangle =\alpha {|0\rangle }_{q_{b}}{|0\rangle }_{q_{c}}+\beta {|1\rangle }_{q_{b}}{|1\rangle }_{q_{c}}.$$

More generally, the four possible Bell outcomes produce the conditional states

(4) $$\begin{array}{cc} |\psi_{BC}^{\left(\Phi^{+}\right)}\rangle & =\alpha {|0\rangle }_{q_{b}}{|0\rangle }_{q_{c}}+\beta {|1\rangle }_{q_{b}}{|1\rangle }_{q_{c}}, \end{array}$$

(5) $$\begin{array}{cc} |\psi_{BC}^{\left(\Phi^{-}\right)}\rangle & =\alpha {|0\rangle }_{q_{b}}{|0\rangle }_{q_{c}}-\beta {|1\rangle }_{q_{b}}{|1\rangle }_{q_{c}}, \end{array}$$

(6) $$\begin{array}{cc} |\psi_{BC}^{\left(\Psi^{+}\right)}\rangle & =\alpha {|1\rangle }_{q_{b}}{|0\rangle }_{q_{c}}+\beta {|0\rangle }_{q_{b}}{|1\rangle }_{q_{c}}, \end{array}$$

(7) $$\begin{array}{cc} |\psi_{BC}^{\left(\Psi^{-}\right)}\rangle & =\alpha {|1\rangle }_{q_{b}}{|0\rangle }_{q_{c}}-\beta {|0\rangle }_{q_{b}}{|1\rangle }_{q_{c}}. \end{array}$$

These post-measurement states are, in general, bipartite entangled states for arbitrary amplitudes α and β. Accordingly, the Bell-basis measurement performed by Alice should not be interpreted as an immediate localization of |X〉 at Bob or Charlie. Instead, it constitutes the elementary routing step of the GHZ MUX: the quantum information becomes encoded in the Bob–Charlie branch in a conditional form determined by Alice’s Bell outcome.

At this stage, the quantum information is not yet localized at a single node, but remains distributed over the Bob–Charlie subsystem. The selection of a specific receiver requires an additional local operation: the qubit belonging to the non-selected branch is measured in the *X* basis. This measurement breaks the bipartite entanglement and projects the selected branch onto the state |X〉 up to a Pauli correction determined jointly by Alice’s Bell outcome and the *X*-basis measurement result. The selected receiver then applies the corresponding Pauli correction based on both Alice’s Bell outcome and the X-basis measurement result, recovering the original state |X〉. This measurement-based localization completes the elementary routing operation in the 1→2 GHZ MUX block.

*Remark on the choice of selection-measurement basis.* The localization step can be implemented using different measurement bases on the non-selected branch. In particular, measurements in the *X* basis preserve a Pauli-frame description of the protocol, while measurements in the *Y* basis also preserve the transmitted quantum information but induce phase corrections outside the Pauli group. For clarity, Table 1 summarizes the effect of different measurement bases in the simplest 1→2 scenario.

**Illustrative example (base case 1→2).** To make the role of the selection measurement explicit, consider the simplest case where Alice performs a Bell-basis measurement and the resulting post-measurement state shared by Bob (*B*) and Charlie (*C*) is

$$\alpha {|0\rangle }_{B}{|0\rangle }_{C}+\beta {|1\rangle }_{B}{|1\rangle }_{C}.$$

This state is bipartite entangled, and the quantum information is not yet localized at either node. Suppose that Charlie is the non-selected receiver. We express his qubit in the *X* basis:

$$|0\rangle =\frac{1}{\sqrt{2}}\left(|+\rangle +|-\rangle \right),\;|1\rangle =\frac{1}{\sqrt{2}}\left(|+\rangle -|-\rangle \right).$$

Substituting, the joint state becomes

$$\frac{1}{\sqrt{2}}\left[{\left(\alpha |0\rangle +\beta |1\rangle \right)}_{B}{|+\rangle }_{C}+{\left(\alpha |0\rangle -\beta |1\rangle \right)}_{B}{|-\rangle }_{C}\right].$$

If Charlie measures in the *X* basis, two outcomes are possible:

- If the outcome is |+〉, Bob’s state becomes |X〉=α|0〉+β|1〉.
- If the outcome is |−〉, Bob’s state becomes Z|X〉=α|0〉−β|1〉.

In both cases, the quantum information is transferred to Bob up to a Pauli correction determined by the measurement outcome. The classical side information generated along the routing process specifies the Pauli frame associated with the active branch. This example shows that the measurement on the non-selected branch does not destroy the quantum state, but rather completes its localization on the selected node.

From the architectural viewpoint of the GHZ MUX, this conditional transfer is the relevant primitive. The classical information generated along the routing process specifies the Pauli frame associated with the active branch, and this information is subsequently used to continue the hierarchical transfer process along the selected route. Thus, the role of classical communication is not to create the quantum transfer by itself, but to coordinate the branch-dependent correction and propagation of the routed state.

For completeness, the Pauli frame associated with each Bell outcome is the standard one inherited from quantum teleportation. These Pauli frames must be updated by incorporating the additional correction induced by the X-basis measurement performed on the non-selected branch:

- $|\Phi^{+}\rangle$: identity frame *I*;
- $|\Phi^{-}\rangle$: phase frame *Z*;
- $|\Psi^{+}\rangle$: bit-flip frame *X*;
- $|\Psi^{-}\rangle$: combined frame XZ.

Therefore, in the 1→2 block, Alice’s Bell measurement injects the unknown state into the first GHZ branch together with the classical side information required to control its subsequent routing. This interpretation is the one that generalizes consistently to the binary GHZ tree considered in the next subsection, where successive relay nodes apply the same transfer principle level by level until the routed state reaches the designated output branch.

### 2.2. Generalization: MUX from 1 to 2^n^

The scheme extends recursively to $2^{n}$ receivers $\left(R_{1},\ldots ,R_{2^{n}}\right)$ by interconnecting $2^{n}-1$ tripartite GHZ states into a binary tree of depth *n*. Each internal node represents an intermediate quantum relay that performs a Bell-basis measurement, propagating the encoded quantum information along one of two branches. For n=2 (four receivers), the structure is defined by:

1. ${|{\mathrm{GHZ}}_{1}\rangle }_{q_{a1}q_{i1}q_{i2}}$: linking Alice ($q_{a1}$) with intermediate nodes $I_{1}$ ($q_{i1}$) and $I_{2}$ ($q_{i2}$).
2. ${|{\mathrm{GHZ}}_{2}\rangle }_{q_{i1}q_{r1}q_{r2}}$: linking $I_{1}$ with receivers $R_{1}$ ($q_{r1}$) and $R_{2}$ ($q_{r2}$).
3. ${|{\mathrm{GHZ}}_{3}\rangle }_{q_{i2}q_{r3}q_{r4}}$: linking $I_{2}$ with receivers $R_{3}$ ($q_{r3}$) and $R_{4}$ ($q_{r4}$).

The initial state of the system is:

$$|\psi_{0}\rangle ={|X\rangle }_{q_{x}}⊗|{\mathrm{GHZ}}_{1}\rangle ⊗|{\mathrm{GHZ}}_{2}\rangle ⊗|{\mathrm{GHZ}}_{3}\rangle .$$

The routing process proceeds through a sequence of *n* Bell-basis measurements that progressively propagate the encoded quantum information along one branch of the binary GHZ tree.

1. Alice measures $\left(q_{x},q_{a1}\right)$, injecting the quantum information into the first GHZ branch associated with intermediate nodes $I_{1}$ and $I_{2}$.
2. The relay node belonging to the selected subtree (either $I_{1}$ or $I_{2}$) performs the next Bell-basis measurement on its incoming qubit and its local GHZ share, thereby propagating the routed state to the next level of the tree.
3. This process continues recursively until the final receiver node $R_{j}$ associated with the selected path is reached.

A total of 2n classical bits convey the sequence of Bell-measurement outcomes produced along the path, together with additional classical information arising from the selection measurements on the non-selected branches, which jointly determine the effective Pauli frame. These classical messages determine the accumulated Pauli frame associated with the routed state. Once the state reaches the final receiver node, the corresponding Pauli correction can be applied to recover the original qubit |X〉. For a network with *k* simultaneous emitters $\left(A_{1},\ldots ,A_{k}\right)$, the scheme scales linearly in GHZ resources, requiring $k(2^{n}-1)$ states while preserving full connectivity across all input–output pairs. This hierarchical switching mechanism thus provides a deterministic yet modular framework for quantum routing based solely on local entanglement operations and classical coordination.

### 2.3. General Formal Representation

The GHZ MUX can be formalized for an arbitrary number of receivers by arranging local tripartite GHZ states in a binary tree of depth *n*, which produces a $1\to 2^{n}$ MUX. The initial global state of the system comprises the input qubit |X〉 held by Alice and a set of $\left(2^{n}-1\right)$ GHZ triples distributed across the internal and terminal nodes of the tree. Formally, the initial state can be written as

(8) $$|\Psi_{0}{\rangle =|X\rangle }_{q_{x}}⊗\overset{2^{n}-1}{\underset{k=1}{⨂}}|{\mathrm{GHZ}}_{k}\rangle ,$$

where each GHZ state has the form $|{\mathrm{GHZ}}_{k}\rangle =(|000\rangle +|111\rangle )/\sqrt{2}.$

At the first level of the tree, Alice performs a Bell-basis measurement on the pair $\left(q_{x},q_{A}\right)$, where $q_{A}$ denotes her share of $|{\mathrm{GHZ}}_{1}\rangle$. Let $m_{1}\in \left\{0,1,2,3\right\}$ represent the outcome of this measurement, corresponding to the four Pauli frames {I,X,Z,XZ}. Conditioned on $m_{1}$, the quantum information encoded in the input state is transferred into the first GHZ branch, together with the Pauli frame information associated with the measurement outcome. At this stage, the quantum information remains distributed over the corresponding branch, rather than localized at a single node. The effective activation of a specific branch is achieved through a subsequent local measurement in the *X* basis on the non-selected subtree. This step removes the residual entanglement with the discarded branch and updates the effective Pauli frame associated with the active branch, incorporating both the Bell outcomes and the selection-measurement outcomes.

More generally, at level *ℓ* of the binary tree, the relay node belonging to the active branch performs a Bell-basis measurement on its local GHZ share $q^{\left(\ell \right)}$ and the incoming qubit $q^{\left(\ell -1\right)}$. Denoting the corresponding measurement outcome by $m_{\ell }$, the state of the system after *ℓ* levels can be represented by

(9) $$|\Psi_{\ell }\left(m_{1},\ldots ,m_{\ell }\right)\rangle =\underset{k>k_{\ell }}{⨂}|{\mathrm{GHZ}}_{k}\rangle ⊗{\left(\sigma \left(m_{\ell }\right)\ldots \sigma \left(m_{1}\right)|X\rangle \right)}_{q_{\mathrm{path}(\ell )}},$$

where $\sigma (m_{j})$ denotes the Pauli operator associated with the *j*-th Bell measurement outcome, and $q_{\mathrm{path}(\ell )}$ represents the effective logical qubit associated with the active branch selected by the first *ℓ* teleportation steps.

The Pauli operator appearing in Equation (9) should be interpreted as an effective operator that accumulates not only the contributions from the Bell-basis measurement outcomes, but also those arising from the *X*-basis selection measurements performed on the non-selected branches at each level. Accordingly, the overall Pauli frame depends jointly on the sequence of Bell outcomes $\left\{m_{1},\ldots ,m_{\ell }\right\}$ and the corresponding selection-measurement outcomes.

After *n* levels, the routed quantum information becomes localized at the receiver node associated with the selected path after the corresponding measurement-induced selection step. The accumulated Pauli frame is then corrected locally, allowing the receiver to recover the original state |X〉.

Equation (9) should be interpreted as describing the effective Pauli frame associated with the active branch after *ℓ* routing steps. At this stage, the quantum information is not yet strictly localized at a single node, but remains distributed over the corresponding branch. The localization into a single qubit is completed only after the measurement-induced selection step that removes the residual entanglement with the non-selected subtree.

This representation highlights that the multiplexer operates through a deterministic cascade of *n* Bell-basis projections that progressively propagate the encoded quantum information along a selected branch of the binary entanglement tree. The structure ensures full input–output connectivity while requiring only local coherence, as each GHZ state participates in exactly one routing decision.

## 3. Comparison of Quantum Devices

The GHZ MUX is compared with two foundational architectures in quantum communication: the quantum switch introduced by Cirac et al. [8] and the quantum router proposed by Pant et al. [10]. The analysis focuses on the routing behavior, coherence requirements, and resource utilization that define the operational trade-offs among these devices.

### 3.1. Quantum Switch

The concept of the quantum switch, introduced by Cirac et al. [8], employs Bell pairs as the fundamental entanglement resource. For instance, the state

$${|\Phi^{+}\rangle }_{ab}=\frac{1}{\sqrt{2}}\left(|00\rangle +|11\rangle \right)$$

serves as the communication channel between two nodes. In a linear network A−B−C, entanglement swapping at the intermediate node *B* enables the indirect entanglement of nodes *A* and *C*. To transfer a quantum state |X〉, the protocol operates as

$$|\psi \rangle ={|X\rangle }_{a}⊗{|\Phi^{+}\rangle }_{bc},\;\mathrm{measure}\;\left(a,b\right)\to \mathrm{send}\;2\;\mathrm{classical}\;\mathrm{bits}\;\mathrm{to}\;c.$$

This architecture features connectivity constrained by the network topology, requiring (N−1) Bell states for *N* nodes and two classical bits per hop. The system operates in a non-blocking fashion, with linear scalability, making it especially suitable for long-distance quantum communication where local entanglement swapping suffices to extend coherence.

### 3.2. Quantum Router

The quantum router extends this paradigm by exploiting multipartite GHZ entanglement to achieve routing in quantum superposition [10]. A representative resource is the four-qubit GHZ state:

$$|{\mathrm{GHZ}}_{4}\rangle =\frac{1}{\sqrt{2}}\left(|0000\rangle +|1111\rangle \right),$$

where each qubit may correspond to a distinct network node. By measuring selected qubits in the *X* basis, effective Bell pairs are dynamically generated between peripheral nodes, establishing entanglement links conditioned on the measurement outcomes. This design allows multiple routing paths to coexist coherently, enabling the simultaneous distribution of quantum information to multiple destinations. However, such capability demands the preservation of global coherence across all nodes sharing the multipartite GHZ state, which poses a significant experimental challenge as system size increases. The Quantum Router thus achieves excellent scalability and flexibility but at the cost of higher coherence requirements and more complex experimental control, aligning with its envisioned role in future quantum internet architectures.

### 3.3. GHZ MUX

The *GHZ MUX* implements deterministic quantum routing from one emitter to one of $2^{n}$ possible receivers using local tripartite GHZ resources arranged in a binary entanglement tree. Rather than relying on global multipartite coherence, the architecture composes small GHZ clusters into a hierarchical structure in which each relay node performs a Bell-basis measurement that propagates the encoded quantum information toward the next level of the selected branch.

In this model, classical communication carries both the sequence of Bell-measurement outcomes and the outcomes of the X-basis selection measurements, jointly defining the accumulated Pauli frame associated with the routed state. After *n* routing stages, the quantum state becomes localized at the receiver associated with the selected branch, where the final Pauli correction recovers the original state |X〉.

Although the scheme is blocking and less scalable than the Quantum Router, it offers a clear advantage in robustness against decoherence and experimental simplicity, since only local GHZ states must be generated and maintained. These characteristics make the GHZ MUX particularly suitable for deterministic quantum communication tasks in near-term network implementations.

### 3.4. Comparison of Routing Behavior

The routing characteristics of these three devices are summarized in Table 2. The GHZ MUX achieves deterministic routing through classical post-selection following Bell-basis measurements, maintaining complete connectivity with only local GHZ entanglement. In contrast, the Quantum Router enables coherent superposition of routes, allowing multiple communication paths to exist simultaneously but requiring global synchronization and coherence maintenance. The Quantum Switch of Cirac et al. relies solely on entanglement swapping between pre-established Bell links, offering low experimental complexity but limited routing flexibility. Overall, Table 2 underscores the intrinsic trade-off between scalability, flexibility, and experimental feasibility among these architectures.

The GHZ MUX does not implement coherent route superposition and therefore should not be interpreted as a quantum router in the fully coherent sense. Instead, it realizes a measurement-driven, deterministic routing mechanism based on local entanglement resources.

### 3.5. Resource Comparison

A quantitative assessment of resource requirements is essential to evaluate the scalability and practical feasibility of different quantum routing architectures. This section compares the GHZ Multiplexer and the Quantum Router in terms of qubit consumption, structural organization, and coherence demands, illustrating how each approach optimizes distinct aspects of quantum network design.

For $2^{n}$ receivers:

- **GHZ MUX**: each receiver follows a unique path within a binary tree of tripartite GHZ states; $N_{\mathrm{qubits}}^{\mathrm{MUX}}=3\left(2^{n}-1\right)+1$ qubits are required, and receiver selection is performed through classical control.
- **Quantum Router**: information is encoded in superposition across all receivers; a single multipartite GHZ state with $N_{\mathrm{qubits}}^{\mathrm{Router}}=2^{n}+2$ qubits suffices, with routing determined quantumly by measuring the control qubit.

The comparison reveals a fundamental trade-off between structural determinism and quantum parallelism. The GHZ MUX constructs a hierarchy of local GHZ states that ensures each receiver is reached through an independent teleportation path. This architecture demands more qubits as the network expands but exhibits strong modularity and robustness to decoherence, since each entangled cluster operates independently. Conversely, the Quantum Router achieves higher efficiency by reducing the number of qubits and enabling route superposition, allowing parallel transmission of quantum information to multiple destinations. However, maintaining the global coherence of a large multipartite GHZ state across a distributed network remains a substantial experimental challenge, as decoherence or loss in any subsystem can disrupt the entire routing process.

In practical terms, the GHZ MUX prioritizes deterministic control and local stability at the expense of scalability, whereas the Quantum Router prioritizes efficiency and quantum parallelism at the expense of coherence robustness. These complementary strategies exemplify two distinct paradigms in quantum network engineering: one oriented toward fault-tolerant, locally coherent operation, and the other toward globally entangled, high-capacity communication.

This comparative assessment suggests that the GHZ MUX represents an intermediate architecture bridging local and global entanglement routing paradigms. Its deterministic control and modular design may enable near-term experimental realizations using existing GHZ-generation platforms, particularly in superconducting or photonic systems where local coherence can be preserved efficiently. Such implementations could serve as practical testbeds for controlled quantum multiplexing at small network scales and inform the design of scalable quantum interconnection protocols.

### 3.6. Fidelity Analysis

A quantitative assessment of the routing fidelity is essential for evaluating the scalability and practical feasibility of the GHZ MUX. Although the architecture operates deterministically at the logical level, the teleportation chain across *n* hierarchical stages introduces cumulative noise originating from (i) the generation of local GHZ states and (ii) imperfect Bell-basis measurements performed at each level of the binary entanglement tree.

Let $F_{\mathrm{GHZ}}$ denote the fidelity of a locally generated tripartite GHZ state with respect to the ideal state $|\mathrm{GHZ}\rangle =(|000\rangle +\left|111\rangle \right)/\sqrt{2},$ and let $F_{\mathrm{BM}}$ denote the fidelity of a Bell-basis measurement performed by a relay node. For a tree of depth *n*, the total number of GHZ states required is $2^{n}-1$, whereas exactly *n* Bell-basis measurements define the route taken by the teleported qubit through the selected branch. Under the standard assumption that errors compound independently across levels, the end-to-end teleportation fidelity is approximated by

(10) $$F_{\mathrm{end}}\left(n\right)\approx F_{\mathrm{GHZ}}^{\;2^{n}-1}\cdot F_{\mathrm{BM}}^{\;n}.$$

This multiplicative model captures the dominant noise contributions in GHZ-mediated hierarchical teleportation: GHZ imperfections accumulate exponentially with network size due to the global preparation of the entanglement structure, whereas Bell-measurement errors contribute linearly with the routing depth. Such behavior is consistent with fidelity analyses reported in multi-hop teleportation and GHZ-assisted routing architectures. The model should be interpreted as a first-order analytical approximation based on independent error contributions (see Refs. [11,12,13] for fidelity analyses in multipartite GHZ-based communication and routing architectures). This exponential dependence highlights a fundamental limitation of the GHZ MUX architecture under the present model: while the routing depth grows linearly with *n*, the number of required GHZ states scales as $2^{n}-1$, leading to a rapid accumulation of imperfections. As a consequence, the architecture is best suited for small-scale quantum networks, particularly in the regime of 4 to 8 output nodes, where the end-to-end fidelity remains within a practically meaningful range.

To illustrate the behavior of Equation (10), we consider representative values observed in recent experiments with superconducting and photonic GHZ generation, namely $F_{\mathrm{GHZ}}=0.98$ and $F_{\mathrm{BM}}=0.99$. Table 3 summarizes the resulting end-to-end fidelity for switches of depth n=1,2,3.

The results highlight a key operational trade-off: while the GHZ MUX achieves deterministic routing with full connectivity and only local coherence requirements, its scalability is ultimately constrained by the exponential accumulation of GHZ-state imperfections. Nonetheless, the fidelity decay observed for depths n≤3 remains within experimentally relevant tolerances, particularly for near-term quantum repeater chains, multiparty key distribution, and GHZ-assisted quantum communication tasks. These findings reinforce the practical feasibility of small- to medium-scale GHZ MUX networks under realistic noise assumptions, and they provide a quantitative foundation for the architectural comparisons developed in the preceding sections.

To complement the analytical estimate in Equation (10), we also performed a numerical simulation incorporating simple but physically meaningful noise contributions along the active routing path. In particular, each routing stage was modeled by combining three effective noise mechanisms: amplitude damping, dephasing, and an imperfect Bell-measurement/correction step represented by a Pauli error channel. The corresponding parameters were chosen as γ=0.01 for amplitude damping, λ=0.005 for dephasing, and p=0.005 for the effective Bell-measurement error probability per stage. These values were selected as conservative low-noise parameters intended to represent a realistic near-term regime without assuming hardware-specific calibration data. The amplitude-damping parameter γ=0.01 captures a small but non-negligible energy-relaxation effect per routing stage, while the dephasing parameter λ=0.005 accounts for moderate phase coherence loss in idle or intermediate qubits. The Bell-measurement error probability p=0.005 was introduced to model residual imperfections in the measurement-and-correction cycle beyond the idealized analytical treatment. Together, these parameters define a minimal realistic-noise scenario suitable for testing whether the analytical expression captures the dominant fidelity trend. Under this model, the numerical fidelities obtained for n=1,2,3 are shown in Table 4. The results remain close to the analytical estimates, indicating that Equation (10) provides a reasonable first-order description of the fidelity decay in the small-scale regime considered here. In particular, both the analytical and numerical results support the interpretation of the GHZ MUX as a practical routing primitive for micro-cluster quantum networks with 4 to 8 output nodes.

The close agreement between analytical and numerical values indicates that the multiplicative model provides a reliable first-order description of fidelity decay in the small-scale regime.

These simulations are intended as a minimal physically motivated validation of the analytical trend, rather than as a hardware-calibrated performance prediction. Their purpose is to verify that the dominant fidelity behavior captured by Equation (10) remains consistent under the inclusion of basic decoherence mechanisms.

## 4. Limitations and Future Work

While the GHZ MUX provides a deterministic and modular mechanism for routing quantum information across a binary teleportation hierarchy, several limitations constrain its scalability and long-distance applicability. These limitations arise primarily from the structure of GHZ entanglement, the cumulative effect of Bell-basis measurements across successive levels, and the exponential growth of required resources.

### 4.1. Local GHZ Fidelity and Decoherence Accumulation

As discussed in the fidelity analysis, the end-to-end performance depends strongly on the quality of the locally generated GHZ states. Since a switch of depth *n* requires $2^{n}-1$ tripartite GHZ resources, any imperfection in these states compounds exponentially, resulting in fidelity decay of the form $F_{\mathrm{end}}\left(n\right)\approx F_{\mathrm{GHZ}}^{2^{n}-1}F_{\mathrm{BM}}^{n}$. This imposes a natural practical limit on the achievable depth, especially when the GHZ states are generated in noisy or lossy platforms such as photonic fiber networks or superconducting circuits with limited coherence times. Although the architecture requires only local coherence—a key advantage over global multipartite GHZ routing—its noise tolerance diminishes as the network grows.

### 4.2. Limited Scalability and Exponential Resource Growth

The entanglement structure of the switch inherently demands an exponential number of GHZ states with respect to network depth. For $2^{n}$ receivers, the switch requires $2^{n}-1$ GHZ nodes arranged in a binary tree. This contrasts with the Quantum Router, which uses a single $\left(2^{n}+2\right)$-qubit GHZ state to serve all receivers. As a result, the GHZ MUX offers improved robustness and modularity but at the cost of reduced scalability. This trade-off positions the device as a near-term architecture suitable for small to medium-sized networks rather than a large-scale quantum internet backbone.

### 4.3. Single-Path Determinism and Absence of Route Superposition

Unlike coherent routing schemes that exploit superposition over multiple paths, the GHZ MUX operates deterministically along a single classically coordinated branch. The sequence of Bell-basis measurements performed along the binary tree progressively propagates the routed state toward a single output node, while classical communication tracks the corresponding Pauli frame. This design eliminates interference effects and simplifies the classical control logic, but it also prevents the simultaneous distribution of quantum information to multiple destinations. Consequently, the architecture is optimized for unicast transmission rather than multicast or superposition-based distribution.

### 4.4. Experimental Considerations

Current experimental platforms vary widely in their ability to generate and maintain high-fidelity tripartite GHZ states. Photonic platforms can produce GHZ states at moderate rates but face loss and heralding inefficiencies, whereas superconducting circuits achieve high fidelities but are limited by connectivity constraints. Additionally, Bell-basis measurements across multiple levels demand precise calibration and synchronization among relay nodes. These practical issues do not undermine the viability of the switch but indicate that near-term implementations will likely be restricted to small depths (n≤3), where both GHZ fidelity and routing reliability remain within experimentally relevant tolerances.

### 4.5. Future Directions

The modular structure of the GHZ MUX suggests several promising research directions. Hybrid architectures combining local GHZ clusters with entanglement swapping could mitigate exponential resource growth. Techniques such as entanglement purification and error-detected teleportation may enhance end-to-end fidelity without altering the core routing mechanism. Finally, integrating the switch into multi-layer quantum network stacks may enable deterministic routing at the application layer while relying on conventional repeater chains for long-distance distribution.

Overall, the GHZ MUX represents a practical intermediate step between deterministic, locally coherent routing and fully coherent, globally entangled quantum networks.

## 5. Conclusions

This work introduced the GHZ multiplexer (GHZ MUX), a hierarchical quantum routing architecture that deterministically transfers an unknown quantum state from a single emitter to one of $2^{n}$ receivers using only locally generated tripartite GHZ states. By organizing these resources into a binary teleportation tree, the architecture achieves full connectivity while maintaining strictly local entanglement requirements and classical feed-forward coordination.

The proposed design contrasts with globally entangled routing approaches such as the Quantum Router [10], which require large multipartite GHZ states shared across the network. Although such architectures provide route superposition and improved qubit efficiency, they impose strong global coherence requirements that remain experimentally challenging. In contrast, the GHZ MUX prioritizes modularity and robustness by composing small GHZ clusters into a deterministic switching structure, making it a promising candidate for near-term implementations of quantum communication networks.

Beyond deterministic routing, the GHZ MUX can also support communication scenarios in which the effective destination of the transmitted quantum state remains hidden from external observers. In particular, the same hierarchical routing structure can be combined with classical control and optional quantum one-time-pad protection to implement a destination-hiding communication primitive. In this setting, the sender determines which leaf ultimately reconstructs the quantum state, while observers without access to the routing record cannot identify the active receiver among a set of candidate nodes.

A formal description of this extension, which we call the Hidden-Secret GHZ-Tree Routing (HS-GTR) protocol, is presented in Appendix A. This construction illustrates how the GHZ MUX architecture can be used not only for hierarchical routing but also as a building block for hidden-destination communication and multi-receiver key-establishment scenarios in quantum networks.

Overall, the GHZ MUX represents a practical intermediate architecture between simple teleportation chains and fully coherent multipartite routing systems. Its modular structure and reliance on local entanglement resources make it particularly suitable for small- to medium-scale quantum networks. Future work may explore hybrid designs that combine GHZ multiplexing with entanglement purification, entanglement swapping, or adaptive routing mechanisms in order to improve scalability and robustness in distributed quantum communication infrastructures.

## Figures and Tables

**Table 1:** Effect of selection measurements on the non-selected branch in the base case 1→2.

Measurement Basis	Outcome	State at Selected Node
*Z*	|0〉 or |1〉	Classical state (superposition destroyed)
*X*	|+〉	|X〉
*X*	|−〉	Z|X〉
*Y*	|+i〉	$S^{†}|X\rangle$
*Y*	|−i〉	S|X〉

**Table 2:** Routing Behavior in Quantum Devices.

Aspect	GHZ MUX	Quantum Router	Quantum Switch
Route Definition	Classically chosen after Bell measurement	Superposition over all possible routes	Fixed, defined by pre-established Bell links
Entanglement Usage	Local tripartite GHZ consumed by teleportation	Global multipartite GHZ maintained until measurement	Local Bell pairs transferred by *entanglement swapping*
Required Coherence	Local	Global	Local per hop
Route Superposition	No	Yes	No
Flexibility	Dynamic selection	Maximum, multiple simultaneous routes	Limited by topology
Experimental Complexity	Moderate	High	Low

**Table 3:** End-to-end teleportation fidelity for representative GHZ and Bell-measurement fidelities.

Depth *n*	$1\;\to \;2^{n}$ MUX Size	$F_{\mathrm{end}}$(*n*)
1	2 receivers	$0.98^{1}\cdot 0.99^{1}\approx 0.97$
2	4 receivers	$0.98^{3}\cdot 0.99^{2}\approx 0.92$
3	8 receivers	$0.98^{7}\cdot 0.99^{3}\approx 0.84$

**Table 4:** Comparison between the analytical fidelity model and numerical simulation with realistic noise contributions along the active routing path.

Depth *n*	$1\;\to \;2^{n}$ MUX Size	Analytical	Numerical
1	2 receivers	≈0.97	≈0.972
2	4 receivers	≈0.92	≈0.926
3	8 receivers	≈0.84	≈0.847

## Data Availability

The original contributions presented in this study are included in the article. Further inquiries can be directed to the corresponding author.

## Appendix A. Hidden-Secret GHZ-Tree Routing

This appendix describes an application of the GHZ MUX architecture to a hidden-state routing scenario. The goal is to move an encrypted quantum state through a binary GHZ-tree structure while preventing external observers, and non-selected branches, from identifying the final carrier node. The protocol does not aim to hide the final carrier from the root node. Instead, the root acts as a trusted authority that prepares the encrypted state and later provides the decryption key to the final carrier.

The construction should be understood as a distributed routing primitive rather than as a composable anonymity protocol. Its hiding property relies on a network model in which the adversary cannot monitor all classical and quantum communications simultaneously.

The routing mechanism can be described in terms of an accumulated Pauli frame, as introduced in Section 2.3, where the sequence of teleportation steps induces a composite Pauli operator corrected at the final receiver. While this representation is compact, it is convenient for distributed implementations to adopt an equivalent formulation in which Pauli corrections are resolved locally at each routing step.

In this Appendix, we use such a local-correction model, in which the routed quantum state is restored to a normalized encrypted form after every level of the tree. This perspective makes explicit the roles of the nodes, the local classical communication required for correction, and the step-by-step propagation of the state. In particular, it yields a modular description in which each level performs the same operation and the state remains recoverable at any stage without requiring knowledge of the full correction history.

This formulation is fully equivalent to the accumulated Pauli-frame description and does not alter the underlying mechanism or performance analysis presented in the main text.

### Appendix A.1. Network Assumptions and Adversarial Model

We consider a large quantum network in which local tripartite GHZ resources can be established between a parent node and two child nodes. The routing structure is represented as a binary tree of prescribed depth. At each level, only local classical communication is required among the parent node, the selected carrier node, and the non-selected dummy node.

The adversary is assumed to have partial visibility of the network. More precisely, the adversary may observe some classical messages and may monitor some network nodes, but is not able to observe the entire network traffic or control all nodes participating in the protocol. Under this assumption, the communication between the final carrier and the root node does not necessarily reveal the final destination to the adversary, because neither the root nor the carrier is assumed to be globally identifiable from the adversary’s partial view.

The protocol therefore provides destination hiding against partial network observers. It does not provide destination hiding against an adversary with global monitoring capability, nor against a coalition that contains all carrier nodes along the active path and the root node.

### Appendix A.2. Node Roles

At each level of the tree, three operational roles are distinguished.

The *parent node* coordinates one local routing step. It holds the current carrier qubit before the step and shares a local tripartite GHZ state with two candidate child nodes.

The *carrier node* is the child selected to continue the propagation of the quantum state. After the local correction step, this node holds the encrypted state in normalized form.

The *dummy node* is the non-selected child. It is measured in the *X* basis in order to remove the residual entanglement with the carrier branch. The dummy node contributes one classical measurement outcome needed for local correction, but it does not need to know the identity of the final carrier node.

This separation is important. The dummy node supplies a correction bit, but the correction information is not sent directly to the final destination. Instead, it is communicated locally to the parent node, which forwards the required local correction information to the selected carrier node.

### Appendix A.3. Input Encryption

Let

|X〉=α|0〉+β|1〉

be the input state. Before injecting the state into the GHZ tree, the root node Alice applies a quantum one-time pad

P(k)∈{I,X,Z,XZ},

where *k* denotes the secret key. The routed state is therefore

$$|\psi_{0}\rangle =P(k)|X\rangle .$$

The purpose of the QOTP is to ensure that the content of the state remains hidden throughout the routing process. Any carrier node that does not know *k* holds only an encrypted version of the state.

### Appendix A.4. Local Routing Step

At a generic level of the tree, suppose that the parent node holds the encrypted state

$$|\psi_{\ell -1}\rangle =P(k)|X\rangle .$$

The parent node shares a tripartite GHZ state with two child nodes. It performs a Bell-basis measurement between its carrier qubit and its local GHZ share. This transfers the quantum information into the two-child branch, but not yet as a localized qubit at a single node.

One child is selected as the next carrier and the other child becomes the dummy. The dummy node measures its qubit in the *X* basis. This measurement breaks the residual entanglement and induces a Pauli correction on the selected carrier branch.

Let $m_{\ell }$ denote the Bell-measurement outcome at level *ℓ*, and let $s_{\ell }$ denote the *X*-basis measurement outcome of the dummy node. Together, these two classical outcomes determine a local Pauli correction

$$C_{\ell }=C\left(m_{\ell },s_{\ell }\right)\in \left\{I,X,Z,XZ\right\}.$$

The dummy sends $s_{\ell }$ to the parent node. The parent node combines this value with its Bell outcome $m_{\ell }$ and sends the corresponding correction instruction to the selected carrier node. The selected carrier applies

$$C_{\ell }^{†}.$$

As a result, the selected carrier node recovers the same encrypted state:

$$|\psi_{\ell }\rangle =P(k)|X\rangle .$$

Thus, the Pauli correction is resolved locally at each level. There is no need to accumulate a global Pauli frame across the tree.

### Appendix A.5. Normalized Propagation Invariant

The protocol maintains the following invariant:

$$|\psi_{\ell }\rangle =P(k)|X\rangle \;\mathrm{for}\;\mathrm{every}\;\mathrm{level}\;\ell .$$

This invariant expresses the main operational difference between the present formulation and a frame-tracking description. Instead of allowing Pauli corrections to accumulate and correcting them only at the final leaf, each local routing step immediately restores the routed qubit to the same encrypted form. Consequently, every carrier node has the same operational state: it holds the encrypted input state and can continue the same routing primitive at the next level.

This makes the protocol modular. Each level performs the same sequence: Bell-basis measurement, dummy-node *X* measurement, local classical communication, local Pauli correction, and propagation of the encrypted state to the next carrier.

### Appendix A.6. Progressive Recoverability

Because the state is restored to P(k)|X〉 at every level, the protocol has a progressive recoverability property. If the routing process is stopped at an intermediate level, the current carrier node can recover the original state provided that Alice supplies the QOTP key *k*.

Indeed, applying $P{\left(k\right)}^{†}$ gives

$$P{\left(k\right)}^{†}P\left(k\right)|X\rangle =|X\rangle .$$

Thus, the state is not only routed but also kept recoverable at each active level. This feature is useful when the desired hiding depth is chosen adaptively, or when the protocol is intentionally terminated before reaching the maximum tree depth.

### Appendix A.7. Final Carrier and Communication with the Root

After the prescribed tree depth is reached, the final carrier node holds

$$|\psi_{n}\rangle =P(k)|X\rangle .$$

At this point, all teleportation-induced corrections have already been resolved locally. The final carrier does not need a global Pauli-frame correction. It only needs the QOTP key from the root node.

The final carrier then communicates with the root node to request or confirm authorization for state recovery. The root node, which acts as the trusted authority, sends the decryption information corresponding to $P{\left(k\right)}^{†}$. After receiving this information, the final carrier applies the inverse QOTP and recovers

|X〉.

This final communication reveals the carrier identity to the root node, but not necessarily to external observers. The hiding claim relies on the assumption that the network is sufficiently large and cannot be fully monitored. Under this assumption, an adversary observing only part of the network cannot reliably identify either the root node or the final carrier node from the final exchange.

### Appendix A.8. Destination Hiding

The destination-hiding property arises from three features.

First, the active carrier changes locally from level to level. Each parent node only needs to know its immediate carrier child and dummy child. It does not need to reveal the full route to the dummy node.

Second, the dummy node contributes only a local *X*-basis measurement outcome. It does not need to know the final destination of the state, and it does not receive information about the complete active path.

Third, the final carrier may be any node in a large network, and the root node itself is not assumed to be globally identifiable by the adversary. Therefore, the final carrier–root communication does not by itself reveal the destination to an adversary with only partial visibility.

The protocol therefore hides the final carrier from external observers and non-selected branches, under the stated partial-monitoring assumption (the protocol does not provide anonymity against a global adversary). It does not hide the final carrier from the root node, since the root provides the decryption key.

### Appendix A.9. Content Hiding

The content-hiding property is provided by the QOTP. Since the routed state is always of the form

P(k)|X〉,

a node that does not know *k* cannot recover the original state. The local Pauli corrections applied during routing do not decrypt the content; they only restore the encrypted state to its normalized form.

Thus, the protocol separates two roles. The GHZ-tree routing mechanism hides and relocates the encrypted quantum state, while the QOTP protects the content until the root node releases the decryption key.

The HS-GTR protocol with local correction proceeds as follows. Alice encrypts the input state using a QOTP and injects the encrypted state into the GHZ tree. At each level, the current carrier node performs a Bell-basis measurement using a local GHZ resource shared with two child nodes. One child is selected as the next carrier and the other as the dummy. The dummy measures in the *X* basis and sends the outcome to the parent. The parent combines this dummy outcome with its Bell outcome and sends the appropriate Pauli correction to the selected carrier. The selected carrier applies the correction and obtains again the encrypted state P(k)|X〉.

After the prescribed depth is reached, the final carrier contacts the root node. The root sends the QOTP decryption information, and the final carrier recovers the original state. Under partial network observability, the destination remains hidden from external observers, while the content remains protected until the root releases the key.
